# Comparison of intravenous versus perineural dexamethasone as a local anaesthetic adjunct for peripheral nerve blocks in the lower limb: A meta-analysis and systematic review

**DOI:** 10.1097/EJA.0000000000002038

**Published:** 2024-07-10

**Authors:** Neel Desai, Suji Pararajasingham, Desire Onwochei, Eric Albrecht

**Affiliations:** From the Department of Anaesthesia, Guy's and St Thomas’ NHS Foundation Trust, London, UK (ND, SP, DO) and Lausanne University Hospital and University of Lausanne, Lausanne, Switzerland (EA)

## Abstract

**BACKGROUND:**

As a local anaesthetic adjunct, the systemic absorption of perineural dexamethasone in the lower limb could be restricted because of decreased vascularity when compared with the upper limb.

**OBJECTIVES:**

To compare the pharmacodynamic characteristics of intravenous and perineural dexamethasone in the lower limb.

**DESIGN:**

Systematic review of randomised controlled trials with meta-analysis.

**DATA SOURCES:**

Systematic search of Central, Google Scholar, Ovid Embase and Ovid Medline to 18 July 2023.

**ELIGIBILITY CRITERIA:**

Randomised controlled trials, which compared the intravenous with perineural administration of dexamethasone as a local anaesthetic adjunct in peripheral nerve blocks for surgery of the lower limb.

**RESULTS:**

The most common peripheral nerve blocks were femoral, sciatic and ankle block. The local anaesthetic was long acting in all trials and the dose of dexamethasone was 8 mg in most trials. The primary outcome, the duration of analgesia, was investigated by all nine trials (*n* = 546 patients). Overall, compared with intravenous dexamethasone, perineural dexamethasone increased the duration of analgesia from 19.54 to 22.27 h, a mean difference [95% confidence interval (CI) of 2.73 (1.07 to 4.38) h; *P* = 0.001, *I*^2^ = 87]. The quality of evidence was moderate owing to serious inconsistency. However, analysis based on the location of the peripheral nerve block, the type of local anaesthetic or the use of perineural adrenaline showed no difference in duration between intravenous and perineural dexamethasone. No differences were shown for any of the secondary outcomes related to efficacy and side effects.

**CONCLUSION:**

In summary, moderate evidence supports the superiority of perineural dexamethasone over intravenous dexamethasone in prolonging the duration of analgesia. However, this difference is unlikely to be clinically relevant. Consideration of the perineural use of dexamethasone should recognise that this route of administration remains off label.

**KEY POINTS**

- As a local anaesthetic adjunct, the systemic absorption of perineural dexamethasone in the lower limb compared with the upper limb could be restricted because of decreased vascularity.
- We performed a systematic review to compare the pharmacodynamic characteristics of intravenous and perineural dexamethasone in the lower limb.
- We included randomised controlled trials that compared the intravenous with perineural administration of dexamethasone as a local anaesthetic adjunct for peripheral nerve blocks in surgery of the lower limb.
- Compared with intravenous dexamethasone, perineural dexamethasone increased the duration of analgesia from 19.54 to 22.27 h, a mean difference of 2.73 h.
- It is unlikely that this difference in the duration of analgesia between perineural and intravenous dexamethasone is sufficient to represent a minimal clinically important difference.

## Introduction

It is common for patients to experience moderate-to-severe pain after surgery on the lower limb. In a prospective observational study of 115 775 patients, calcaneal open reduction led to the highest average pain severity on the first postoperative day.^1^ The development of uncontrolled pain can result in decreased patient satisfaction, increased pulmonary and cardiac complications, and delayed ambulation and has been associated with chronic pain.^2^ Opioids are frequently administered for analgesia but may themselves produce side effects such as respiratory depression, secondary hyperalgesia, constipation, nausea and vomiting and pruritus.^3^ They have also been related to problems such persistent opioid use and abuse.^4^ To improve the provision of pain relief and reduce the incidence of opioid-related undesirable reactions, anaesthetists often use a single-shot peripheral nerve block as part of their analgesic regimen.^5^

However, single-shot peripheral nerve block with long-acting local anaesthetic alone is associated with a duration of analgesia that does not match the duration of postoperative noxious stimuli. This limited duration of pain relief, estimated to be 8 to 12 h,^6,7^ can lead to rebound or residual pain on resolution of the sensory blockade, and a consequent increased consumption of opioids, overnight sleep disturbance and difficulties in compliance with elements of enhanced recovery and physiotherapy protocols.^8^ Local anaesthetic adjuncts have been defined as the simultaneous administration of one or more drugs around the peripheral nerve or plexus, into a fascial plane, or systematically by intravenous injection^9^ and represent a pragmatic and technically simple analgesic strategy to increase the duration of analgesia and overcome some of the problems of single-shot peripheral nerve block.^10^

A recent network meta-analysis of 100 trials and 5728 patients compared dexamethasone and dexmedetomidine as local anaesthetic adjuncts, administered either perineurally or intravenously, along with long-acting local anaesthetics in supraclavicular brachial plexus block. Whole group analysis, ignoring the route of administration of the adjuncts, found an increased duration of analgesia and sensory blockade in the dexamethasone group.^11^ When the two routes of administration of dexamethasone were compared, they were comparable in increasing the duration of sensory blockade (477 vs. 411 min) and duration of analgesia (478 vs. 518 min) when administered via the intravenous and perineural routes, respectively. It is possible that the similarity in these durations between intravenous and perineural dexamethasone may be a reflection of the latter's significant systemic absorption due to the vascularity of the upper limb. Once in the systemic circulation, dexamethasone can reduce inflammation. Compared with the upper limb, the lower limb is relatively less vascular and hence the systemic absorption of perineural dexamethasone in this particular anatomical site might be relatively restricted.^12^

Compared with the upper limb, few trials have investigated the effect of dexamethasone as a local anaesthetic adjunct in peripheral nerve blocks of the lower limb,^13,14^ and it is unclear if the efficacy of intravenous and perineural administration of dexamethasone remains similar in the lower limb. No systematic review to date has specifically studied the role of intravenous and perineural dexamethasone in the lower limb. In view of this, our aim was to perform a systematic review to evaluate the efficacy and side effects of intravenous relative to perineural dexamethasone in the context of peripheral nerve block for surgery on the lower limb.

## Methods

In the conduct of our meta-analysis and systematic review, we adopted the recommendations from the Preferred Reporting Items for Systematic Reviews and Meta-Analyses (PRISMA)^15^ and registered it on the International Prospective Register of Systematic Reviews (PROSPERO) (CRD 42023442453).

### Search strategy, eligibility criteria and selection process

The following databases, Central, Google Scholar, Ovid Embase and Ovid Medline, were searched from their inception to 18 July 2023 for relevant trials. Controlled vocabulary terms and text words associated with the components of this review were chosen, and these included ‘peripheral nerve block’ and ‘dexamethasone’ (Supplemental Digital Content 1). Citations that were retrieved from the search strategy were entered into a reference management program, Rayyan (Qatar Computing Research Institute, 2016, Doha, Qatar),^16^ and duplicate citations were removed. The title and abstract of the remaining citations were screened for eligibility by two authors (EA and ND), and the full texts of potentially eligible citations were subsequently assessed for inclusion. Only randomised controlled trials comparing the intravenous with perineural administration of dexamethasone as a local anaesthetic adjunct in peripheral nerve blocks for surgery of the lower limb were eligible for inclusion. Discrepancies between the two authors in the decision to include trials were resolved by involvement of a third author (DO). The reference lists of included trials were manually searched for those not identified by the previously described search strategy.

### Risk of bias

Once all the trials included in the meta-analysis were determined, the risk of bias, a measure of their methodological quality, was assessed for those published in full using the risk of bias 2 (RoB 2) tool by two authors (DO and SP).^17^ The following five domains of bias were evaluated: the bias arising from the randomisation process; bias due to deviations from intended interventions; bias due to missing outcome data; bias in measurement of the outcome and the bias in selection of the reported result. Overall findings were summarised for the individual trials as indicating low risk of bias, some concerns or high risk of bias.

### Characteristics of trials and data extraction

Characteristics extracted from each trial included: the disorder necessitating surgery; nature of surgery; use of a surgical tourniquet; type of peripheral nerve block; technique for peripheral nerve block; main mode of anaesthesia; intraoperative systemic analgesia and the postoperative systemic analgesia. The primary outcome was the duration of analgesia, and this was defined as the time interval between the performance of the peripheral nerve block or onset of sensory blockade and the time of the first analgesic request or initial reporting of pain. Data related to the following secondary outcomes were obtained: the onset of sensory and motor blockade; duration of motor blockade; pain score at rest and on movement at 6, 12, 24 and 48 h; cumulative intravenous morphine consumption at 6, 12, 24 and 48 h; incidence of nausea and vomiting at 24 h and in hospital; incidence of postoperative hyperglycaemia, infection and neurological complications; and the patient satisfaction. Continuous outcomes were extracted as mean ± standard deviation and, as per the recommendations of the Cochrane Collaboration, the median was assumed to be equal to the mean and the standard deviation was estimated to be the interquartile range/1.35 or the range/4.^18^ Dichotomous outcomes were extracted as number of occurrences. Numerical values of data that were presented in only graphical format were derived by the digitisation of the plot with Plot Digitizer (Version 2.1, Free Software Foundation, 2015, Boston, USA). The details of missing or unclear methodology or data were requested up to three times from the corresponding authors of the trials.

### Statistical analysis

Data were input from a standardised form in Microsoft Excel (Microsoft Corp, Redmond, Washington, USA) to Review Manager (Version 5.3, The Nordic Cochrane Centre, 2014, Copenhagen, Denmark), and an outcome was only subjected to meta-analysis if it was reported by two or more randomised controlled trials. Statistical heterogeneity, *I*^2^, owing to clinical or methodological diversity, was calculated for each outcome. The predetermined thresholds for low, moderate and high levels of heterogeneity were 25 to 49, 50 to 74% and more than or equal to 75%.^19^ In the case of low heterogeneity, it was assumed that the true effect of the intervention was the same in every trial, and a fixed effect model was selected to represent the best estimate of the intervention effect. In the event of moderate or high heterogeneity, it was assumed that the effect of the intervention was not the same in every trial but followed some distribution, and the DerSimonian and Laird random effects model was selected to signify the average intervention effect. For continuous outcomes, the inverse variance method was used in which the weight allocated to every trial was the inverse of the variance of the effect estimate, leading to the computation of a weighted mean difference (95% CI). For dichotomous outcomes, the Mantel–Haenzel method was used, resulting in the computation of a risk ratio (95% CI). Subgroup analyses were performed for the quality of trial, location of peripheral nerve block, type of local anaesthetic and the use of perineural adrenaline. All tests were two-tailed and undertaken at the 5% statistical significance level.

### Quality of evidence

The quality of evidence for every outcome was assessed with the Grading of Recommendations Assessment, Development and Evaluation (GRADE) system.^20^ If serious limitations were present in the domains of risk of bias, inconsistency, indirectness, imprecision and publication bias, then the quality of evidence was downgraded. Publication bias was only considered if 10 or more trials reported a particular outcome, and was evaluated by reference to a funnel plot and the performance of Duval and Tweedie's trim and fill test and Egger's linear regression test with Comprehensive Meta-Analysis (Version 3.3, Biostat, 2014, New Jersey, USA). In Duval and Tweedie's trim and fill test, the smaller trials producing funnel plot asymmetry were removed and the omitted trials and their missing counterparts were replaced.

## Results

**Fig. 1:**
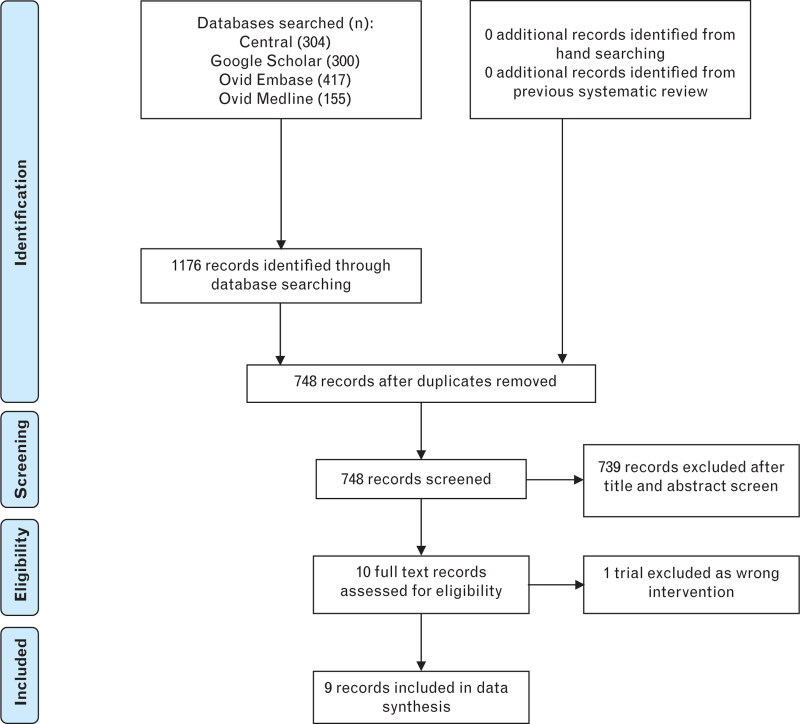
PRISMA flow diagram summarising the retrieved, included and the excluded randomised controlled trials.

**Fig. 2:**
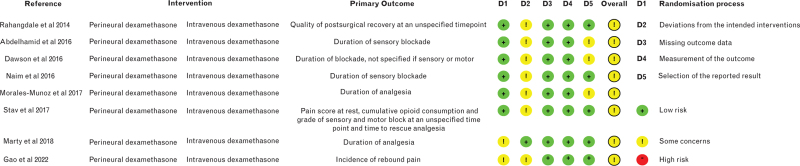
Risk of bias assessment of included trials using the Cochrane's Collaboration's tool.

In all, we included nine trials that investigated the effect of local anaesthetic adjuncts for peripheral nerve block in surgery for the lower limb in 546 patients.^13,14,21–27^ The details of the screening process are illustrated in Fig. 1 and the findings of the risk of bias assessment are presented in Fig. 2. There were concerns for all trials, particularly because of the presence of deviations from the intended interventions.

PRISMA, Preferred Reporting Items for Systematic Reviews and Meta-Analyses.

?, unclear risk; -, high risk; + low risk.

### Characteristics of trials

Characteristics of the trials are listed in Table 1. The nature of the surgery was lower limb in general in one trial,^22^ on the knee in three trials,^21,23,24^ distal to the knee in one trial^26^ and on the ankle and foot in four trials.^13,14,25,27^ The most common peripheral nerve blocks were the femoral and sciatic nerve block, conducted in three trials,^22,26,27^ and the ankle block, provided in two trials.^13,25^ Ultrasound was used to perform the peripheral nerve block in five trials,^13,14,24,25,27^ whereas the techniques utilised to identify the nerve were landmark and neurostimulation, neurostimulation and ultrasound and not specified in two,^21,22^ one^23^ and one trial,^26^ respectively. With regard to the constituents of the solution administered in the peripheral nerve blocks, bupivacaine was given in five trials^14,21,22,24,26^ and ropivacaine in four trials.^13,23,25,27^ The dose of perineural dexamethasone was 8 mg in most trials^13,14,21–24,26^ and was lower (4 mg) in one trial,^25^ and higher (10 mg) in another trial.^27^ In addition to intravenous or perineural dexamethasone, perineural adrenaline was used in two trials.^14,24^ The mode of anaesthesia was dependent on general or spinal anaesthesia and not the peripheral nerve block in three trials,^23,24,27^ and general anaesthesia was only utilised if either the onset of regional anaesthesia was delayed or the regional anaesthesia was patchy or inadequate in two trials.^13,22^

**Table 1:** Characteristics of the included trials

Reference	Group	Nature of surgery / use of tourniquet	Type of peripheral nerve block	Technique for peripheral nerve block	Main mode of anaesthesia	Perioperative systemic analgesia
Rahangdale *et al.*, 2014^14^	Intravenous dexamethasone (*n* = 23) Perineural dexamethasone (*n* = 27)	Ankle and foot surgery Use of surgical tourniquet in 79.3% of cases	Sciatic nerve block Saphenous nerve block	Sciatic nerve block: ultrasound-guided and unilateral single-shot injection: 0.45 ml kg^−1^ bupivacaine 0.5% to a maximum volume of 40 ml with either intravenous or perineural dexamethasone 8 mg and perineural adrenaline 3.33 μg ml^−1^. Saphenous nerve block: not specified.	Sedation and regional anaesthesia	Intraoperative: intravenous fentanyl Postoperative: PRN paracetamol and hydromorphone of unspecified route
Abdelhamid *et al.*, 2016^21^	Intravenous dexamethasone (*n* = 20) Perineural dexamethasone (*n* = 20)	Arthroscopic knee surgery Use of surgical tourniquet	Lumbar plexus block Sciatic nerve block	Lumbar plexus block: landmark and neurostimulation-guided and single-shot injection: 20 ml bupivacaine 0.5% with either intravenous or perineural dexamethasone 8 mg, but timing of intravenous dexamethasone in relation to tourniquet inflation not specified Sciatic nerve block: landmark and neurostimulation-guided and single-shot injection: 10 ml bupivacaine 0.5%	Sedation and regional anaesthesia	Intraoperative: none Postoperative: PRN paracetamol
Dawson *et al.*, 2016^13^	Intravenous dexamethasone (*n* = 30) Perineural dexamethasone (*n* = 30)	Metatarsal osteotomy Use of surgical tourniquet not specified	Ankle block Saphenous and sural nerves not blocked	Ultrasound-guided and single-shot injection: 20 ml ropivacaine 0.75% with either intravenous or perineural dexamethasone 8 mg	Regional anaesthesia General anaesthesia if onset of regional anaesthesia was delayed	Intraoperative: none Postoperative: PRN oral paracetamol, tramadol and oxycodone
Naim *et al.*, 2016^22^	Intravenous dexamethasone (*n* = 21) Perineural dexamethasone (*n* = 21)	Lower limb vascular surgery Use of surgical tourniquet not specified	Femoral nerve block Sciatic nerve block	Femoral and sciatic nerve block: landmark and neurostimulation-guided and single-shot injection. For each peripheral nerve block, 20 ml bupivacaine 0.5% with either intravenous or perineural dexamethasone 8 mg	Sedation and regional anaesthesia General anaesthesia if regional anaesthesia was patchy or inadequate	Intraoperative: none Postoperative: PRN intramuscular diclofenac and intravenous paracetamol and pethidine
Morales-Muñoz *et al.*, 2017^23^	Intravenous dexamethasone (*n* = 27) Perineural dexamethasone (*n* = 27)	Total knee arthroplasty Use of surgical tourniquet not specified	Femoral nerve block	Neurostimulation and ultrasound-guided and single-shot injection: 20 ml ropivacaine 0.5% with either intravenous or perineural dexamethasone 8 mg	Spinal anaesthesia	Intraoperative: none Postoperative: regular intravenous infusion of metamizole and patient-controlled analgesia with morphine
Stav *et al.*, 2017^24^	Intravenous dexamethasone (*n* = 30) Perineural dexamethasone (*n* = 30)	Total knee arthroplasty Use of surgical tourniquet not specified	Femoral nerve block Obturator nerve block Lateral femoral cutaneous nerve block Popliteal sciatic nerve block	Femoral, obturator, lateral femoral cutaneous and popliteal sciatic nerve block: ultrasound-guided and single-shot injection. For femoral, obturator and popliteal sciatic nerve block, 5, 10 and 15 ml 0.5% bupivacaine with perineural adrenaline 5 μg ml^−1^, respectively For lateral femoral cutaneous nerve block, 5 ml lidocaine 1% Either intravenous or perineural dexamethasone 8 mg in total	General anaesthesia	Intraoperative: intravenous fentanyl Postoperative: PRN paracetamol, metamizole, diclofenac, ketorolac, tramadol, meperidine, morphine and oxycodone of unspecified route
Marty *et al.*, 2018^25^	Intravenous dexamethasone (*n* = 50) Perineural dexamethasone (*n* = 50)	Metatarsal osteotomy Use of surgical tourniquet	Ankle block	Ultrasound-guided and single-shot injection: 30 ml ropivacaine 0.375% with either intravenous dexamethasone 10 mg or perineural dexamethasone 4 mg, but timing of intravenous dexamethasone in relation to tourniquet inflation not specified	Sedation and regional anaesthesia	Intraoperative: not specified Postoperative: regular paracetamol and ketoprofen of unspecified route and PRN oral tramadol
Abdelaziz *et al.*, 2020^26^	Intravenous dexamethasone (*n* = 25) Perineural dexamethasone (*n* = 25)	Lower limb surgery distal to knee Use of surgical tourniquet not specified	Femoral nerve block Sciatic nerve block	Femoral and sciatic nerve block: Method of localisation of nerve not specified For each peripheral nerve block, 20 ml bupivacaine 0.5% with either intravenous or perineural dexamethasone 8 mg	Not specified	Intraoperative: not specified Postoperative: not specified
Gao *et al.*, 2022^27^	Intravenous dexamethasone (*n* = 45) Perineural dexamethasone (*n* = 45)	Ankle open reduction and internal fixation of fracture Use of surgical tourniquet	Femoral nerve block Popliteal sciatic nerve block	Femoral and popliteal sciatic nerve block: Ultrasound-guided and single-shot injection For each peripheral nerve block, 20 ml ropivacaine 0.375% with either intravenous or perineural dexamethasone 10 mg, but timing of intravenous dexamethasone in relation to tourniquet inflation not specified	General anaesthesia	Intraoperative: intravenous sufentanil Postoperative: regular intravenous patient-controlled analgesia with sufentanil and PRN intravenous ketorolac

*n*, number of patients in that arm of a study; PRN, as required.

### Primary outcome

**Fig. 3:**
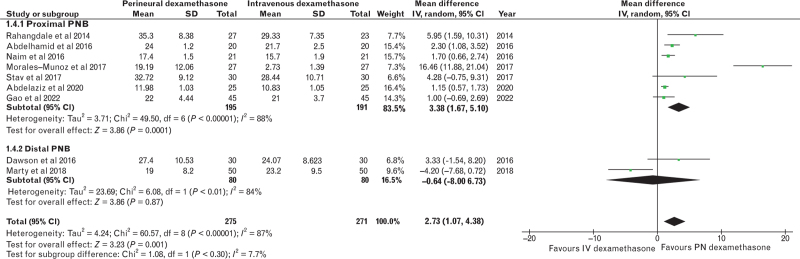
Forest plot of the duration of analgesia.

Our primary outcome, the duration of analgesia, was studied by nine trials (*n* = 546).^13,14,21–27^ In comparison with intravenous dexamethasone, perineural dexamethasone increased the duration of analgesia from 19.54 to 22.27 h, a mean difference (95% CI) of 2.73 (1.07 to 4.38) h; *P* = 0.001, *I*^2^ = 87; Fig. 3). Leave-one-out sensitivity analysis demonstrated no change in the superiority of perineural over intravenous dexamethasone when any one of the trials was removed. The mean difference (95% CI) varied from 1.54 (0.50 to 2.58) h with the exclusion of Morales-Muñoz *et al.*^23^ to 3.39 (1.73 to 5.04) with the omission of Marty *et al.*^25^ Subgroup analysis based on the location of the peripheral nerve block (proximal, defined as far as and including the knee versus distal, that is past the knee; *P* = 0.30), type of local anaesthetic (bupivacaine versus ropivacaine; *P* = 0.56) or use of perineural adrenaline (*P* = 0.13). revealed no differences between intravenous and perineural dexamethasone. No differences were shown for any of the secondary outcomes that are listed in Table 2. The quality of evidence was moderate owing to the presence of serious limitations for the primary outcome and mostly low or moderate for the secondary outcomes as listed in Table 3.

For each trial, the square depicts the risk ratio and the horizontal lines either side of it represent the 95% CI. The summary result is presented as a diamond. CI, confidence interval; SD, standard deviation.

**Table 2:** Meta-analysis of secondary outcomes

		Number of patients				
Outcomes	Number of trials	Perineural	Intravenous	Effect size (95% CI)	*I*^2^ (%)	*P* value
Onset of sensory blockade (min)^21,22,26^	3	87	87	−1.12 (−4.65 to 2.42)	93	0.53
Onset of motor blockade (min)^21,22,26^	3	87	87	−0.08 (−2.24 to 2.26)	78	0.95
Duration of motor blockade (h)^21,22^	2	41	41	2.24 (−1.19 to 5.67)	95	0.20
Pain at rest at 12 h (0 to 10)^24,27^	2	75	75	0 (−0.04 to 0.04)	0	1
Pain at rest at 24 h (0 to 10)^14,23,24,27^	4	129	125	−1.32 (−3.20 to 0.55)	94	0.17
Pain on movement at 24 h (0 to 10)^14,24^	2	57	53	−1.50 (−5.29 to 2.29)	83	0.44
Pain at rest at 48 h (0 to 10)^14,23,24,27^	4	129	125	−0.12 (−1.53 to 1.29)	93	0.87
Pain on movement at 48 h (0 to 10)^14,24^	2	57	53	0.23 (−1.13 to 1.59)	0	0.74
Cumulative intravenous morphine equivalent consumption at 24 h (mg)^13,14,23,24^	4	114	110	−7.17 (−15.66 to 1.31)	90	0.10
Cumulative intravenous morphine equivalent consumption at 48 h (mg)^23–25^	3	107	107	−9.33 (−31.63 to 12.98)	96	0.41
Rate of in hospital postoperative nausea and vomiting (%)^23,25,27^	3	122	122	1.73 (0.86 to 3.48)	38	0.13
Patient satisfaction (0 to 10)^14,21,27^	3	92	88	0 (−0.05 to 0.06)	0	0.94

Values are mean difference, standardised mean difference or risk ratio (95% confidence interval).

**Table 3:**
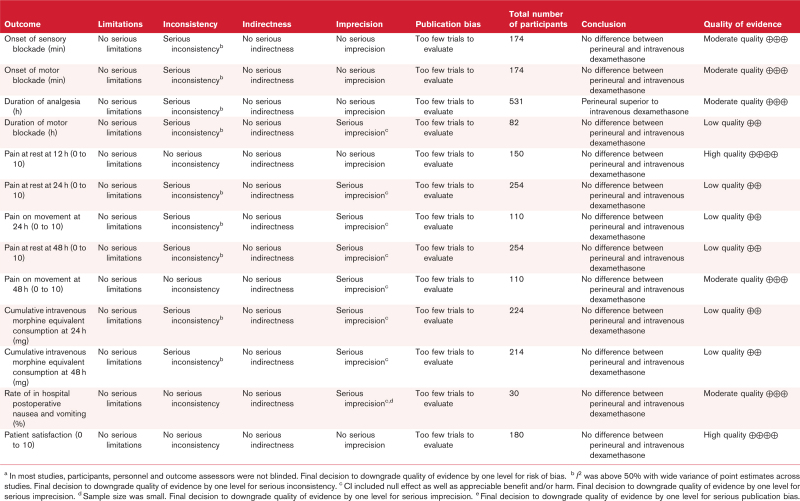
Grading of Recommendations Assessment, Development and Evaluation (GRADE) quality of evidence assessment for each outcome

### Secondary outcomes

Data related to the incidence of postoperative hyperglycaemia, infection and neurological complications were not subjected to meta-analysis because of insufficient information, or significant heterogeneity in their definitions. In one trial, differences were not found in the incidence of postoperative hyperglycaemia^23^ and in two trials, no postoperative infection was revealed.^23,27^ Neurological complications were defined in an inconsistent manner and measured at a varying time point. Five trials, in which the definition and time point varied from no paraesthesia, numbness or weakness at the time of hospital discharge to no neurological complications in the first 48 h, reported no observed neurological complications.^22–25,27^ In one trial, even though no differences were shown between intravenous and perineural dexamethasone, patients said they had paraesthesia and dysaesthesia on the first and second postoperative day as well as at 2 weeks, and numbness was reported at 2 weeks and on the first month.^14^ Their neurological symptoms, however, did not persist beyond the eighth week and resolved without intervention.

## Discussion

In our meta-analysis, compared with intravenous dexamethasone, perineural dexamethasone as a local anaesthetic adjunct in the setting of peripheral nerve block for surgery on the lower limb increased the duration of analgesia from 19.54 to 22.27 h, a mean difference of 2.73 h. The quality of evidence was moderate. No differences were demonstrated between intravenous and perineural dexamethasone with regard to other indices of efficacy and side effects, including the duration of motor blockade and cumulative intravenous morphine equivalent consumption at 24 h. Perineural dexamethasone was not shown to increase the incidence of postoperative hyperglycaemia, infection and neurological complications relative to intravenous dexamethasone.

The minimal clinically important difference (MCID) is the smallest difference in the domain of interest that patients perceive as beneficial, and which would mandate, in the absence of troublesome side effects and excessive costs, a change in patient management.^28^ The MCID can bridge the gap between statistical significance and clinical meaningfulness,^29^ but, to the knowledge of the authors, the MCID for the duration of analgesia, has not been determined. If the MCID were to be set at 20% or greater, as is common in the absence of evidence in many randomised controlled trials, then the statistically significant increase in the duration of analgesia with perineural dexamethasone from 19.54 to 22.27 h, a mean difference of 2.73 h, is still less than 24 h and leads to a subthreshold difference of 14%, disputing the clinical relevance of this result.

Evidence has accumulated to indicate that the mechanism of action of dexamethasone when administered as a local anaesthetic adjunct is systemic rather than perineural in nature.^30,31^ In one pharmacokinetic study, the intravenous or perineural use of dexamethasone in conjunction with supraclavicular brachial plexus block for surgery on the upper limb led to a comparable maximum plasma concentration, time to achieving the average maximum concentration of dexamethasone and area under the concentration curve.^31^ These findings suggested that most of the perineural dexamethasone was absorbed into the systemic circulation through the blood vessels in the relevant anatomical locations of the upper limb. Further, in a randomised controlled trial of the ulnar nerve block in healthy volunteers not scheduled for surgery, neither intravenous nor perineural dexamethasone resulted in a prolongation of the sensory block relative to isotonic saline.^30^ This was likely to be because of the fact that there was an absence of inflammation in such patients, and the lack of a target site of action for dexamethasone.

In this systematic review, the superiority of perineural over intravenous dexamethasone with regard to the duration of analgesia may represent the influence of a decreased systemic absorption in the relatively less vascular anatomical location of the lower limb.^32^ The reduced vascularity of the lower limb compared with the upper limb was thought to be responsible for the increased incidence of local anaesthetic systemic toxicity subsequent to the performance of peripheral nerve block on the upper limb in an international and multicentre registry.^33,34^ If perineural dexamethasone were to be slowly rather than well absorbed into the systemic circulation once injected for surgery on the lower limb relative to the upper limb, it would then have, in the opinion of the authors, either a depot effect, providing an increased duration of analgesia by maintaining the minimally effective plasma concentration for a longer period of time, or have an opportunity to exert its effect on its perineural rather than systemic target site. Dexamethasone can interact with glucocorticoid receptors on the neuronal membrane, increasing the expression of inhibitory potassium channels and thereby decreasing the excitability of nociceptive and unmyelinated C fibres.^35^ No differences were found in any of the secondary outcomes, and this may reflect the paucity of evidence with respect to the use of dexamethasone as a local anaesthetic adjunct for peripheral nerve block in surgery on the lower limb. Compared with the primary outcome, which was described by all of the included trials, the secondary outcomes were variably reported by only two to four of them, increasing the possibility of false-negative errors.^36^

Concerns have been raised as regards the myotoxic and neurotoxic potential of dexamethasone when administered via the perineural route.^35^ Of interest, the perineural use of dexamethasone is off-label^37^: it is not licensed for administration via the perineural route by the European Medicines Agency, Food and Drug Administration and the Medicines and Healthcare product Regulatory Agency. However, the evidence to date indicates that perineural dexamethasone is not neurotoxic.^38–40^ In one in-vitro study, dexamethasone decreased the cytotoxic effect of bupivacaine on mouse neuroblastoma cells,^38^ and in another in-vitro study, dexamethasone at high dose and when co-administered with ropivacaine did not have an increased neurotoxic effect compared with ropivacaine alone on rat dorsal root ganglia.^39^ Moreover, in an in-vivo study, the co-administration of dexamethasone and bupivacaine for sciatic nerve block in rats did not lead to histopathological or neurobehavioural effects.^40^ In the trials included in this meta-analysis, either no differences were revealed between perineural and intravenous dexamethasone with respect to the incidence of neurological complications or no neurological complications were reported. Importantly, in a series of greater than 2000 intrathecal injections of dexamethasone for posttraumatic visual disturbance, no neurological sequelae were shown to occur.^41^ However, it has been estimated that more than 16 000 patients would be required to uncover a doubling of the low baseline rate of nerve injury,^42^ and this systematic review was significantly underpowered to investigate such a difference.

Given the possible different absorption characteristics of dexamethasone when administered via the perineural route in the lower and upper limb, our findings were not inconsistent with the results of previous meta-analyses and systematic reviews.^11,43–45^ The administration of intravenous and perineural dexamethasone as a local anaesthetic adjunct was evaluated in three meta-analyses that included peripheral nerve blocks for the upper and lower limbs.^43–45^ These found the duration of analgesia to be prolonged with the use of perineural compared with intravenous dexamethasone. It has been suggested that the pooling of peripheral nerve blocks for the upper and lower limbs may have influenced the direction of the overall intervention effect in favour of perineural dexamethasone.^11^ The administration of intravenous and perineural dexamethasone as a local anaesthetic adjunct was examined in one meta-analysis, which only included peripheral nerve blocks for the upper but not lower limb.^11^ This showed the duration of analgesia as similar with the use of either intravenous or perineural dexamethasone, potentially owing to the significant systemic absorption of the latter in the upper limb.

### Limitations

Some limitations serve to restrict the interpretation of the findings. First, the number of trials and patients were limited. Second, heterogeneity was present in the characteristics of the trials. This included the nature of surgery, location of peripheral nerve block, type of local anaesthetic and the main mode of anaesthesia. Subgroup analyses, however, did not find differences because of the location of peripheral nerve block or type of local anaesthetic. Of note, all the trials used long-acting local anaesthetic and therefore the results should not be generalised to situations where the administration of short-acting local anaesthetic is appropriate. The evidence would be enhanced with the results of additional trials that use a consistent methodology to provide further characterisation of the clinical improvement with perineural compared with intravenous dexamethasone. In particular, trials which study the role of perineural versus intravenous dexamethasone in the absence of neuraxial or general anaesthesia would strengthen the relationship between dexamethasone as a local anaesthetic adjunct and the duration of analgesia. Third, in one trial, the dose of dexamethasone was different depending on the route of administration.^25^ Fourth, the analgesic effectiveness of dexamethasone may vary depending on other factors, such as the concomitant administration of nonsteroidal anti-inflammatory drugs or the timing of intravenous dexamethasone in relation to the use of a surgical tourniquet. The administration of nonsteroidal anti-inflammatory drugs is important as intravenous dexamethasone and perineural dexamethasone, once absorbed into the systemic circulation, is thought to reduce systemic inflammation. The use of a surgical tourniquet is relevant, as the mechanism of action of systemic dexamethasone may be hindered if it is administered subsequent to tourniquet inflation. Inadequate data were available to facilitate the analysis of the influence of these factors. Fifth, heterogeneity was present in the definition of some of the outcomes. Neurological complications, for instance, were inconsistently defined, and also measured at a varying time point. Sixth, trials were limited by the risk of bias and the quality of outcomes varied as a result of serious inconsistency and/or imprecision.

## Conclusion

In conclusion, our meta-analysis and systematic review showed that perineural dexamethasone was superior to intravenous dexamethasone in prolonging the duration of analgesia with a moderate quality of evidence. It is unlikely, however, that the magnitude of difference between perineural and intravenous dexamethasone was sufficient to represent the MCID. Secondary outcomes related to other markers of efficacy and side effects were not influenced. Consideration of the perineural use of dexamethasone should recognise that this route of administration remains off label.

## Supplementary Material

Supplemental Digital Content
