# Supplementary material for: Comparison of intravenous versus perineural dexamethasone as a local anaesthetic adjunct for peripheral nerve blocks in the lower limb: A meta-analysis and systematic review
Source: Eur J Anaesthesiol. 2024 Jul 10;41(10):749–59. doi: 10.1097/EJA.0000000000002038 (PMC11377050; doi:10.1097/EJA.0000000000002038)
Supplement: Supplemental Digital Content [file ejanet-41-749-s001.docx]

**Perineural versus intravenous dexamethasone in peripheral nerve blocks** RE_Nerve_Block_Dexamethasone_2023_0728.docx

**Recherche réalisée à l'attention de :** Eric Albrecht

**Finalité de la recherche :** Systematic review

**Recherche effectuée par :** Cécile Jaques

Medical Library, Lausanne University Hospital and University of Lausanne, Rue du Bugnon 46, 1011 Lausanne, Switzerland

**Recherche révisée par :** Jolanda Elmers

**Date finale des recherches : 18.07.2023**

**Table des matières**

[1. Question de recherche 2](#_Toc140587786)

[PICO framework 2](#_Toc140587787)

[2. Recherche préliminaire de revues systématiques et/ou scoping reviews 2](#_Toc140587788)

[3. Sources de données exploitées 3](#_Toc140587789)

[4. Vocabulaire 4](#_Toc140587790)

[5. Stratégies de recherche 7](#_Toc140587791)

[Medline ALL Ovid 7](#_Toc140587792)

[Embase.com 7](#_Toc140587793)

[Cochrane Central Register of Controlled Trials Wiley 9](#_Toc140587794)

[Google Scholar 9](#_Toc140587795)

[6. Résultats 10](#_Toc140587796)

# Question de recherche

L’administration de dexaméthasone par voie périneurale est-elle plus efficace que par voie systémique lorsque utilisée comme adjuvant aux anesthésiques locaux lors de blocs nerveux périphériques ?

## PICO framework

**P**opulation: blocs nerveux périphériques

**I**ntervention: dexaméthasone par voie périneurale

**C**omparison: dexaméthasone par voie systémique

**O**utcome: contrôle de la douleur

# Recherche préliminaire de revues systématiques et/ou scoping reviews

Revues systématiques identifiées :

34963269 **29164528** **28854551** 28252523

[Lien vers les articles dans PubMed](https://pubmed.ncbi.nlm.nih.gov/?term=34963269+29164528+28854551+28252523)

- Tan ESJ, Tan YR, Liu CWY. Efficacy of perineural versus intravenous dexamethasone in prolonging the duration of analgesia when administered with peripheral nerve blocks: a systematic review and meta-analysis. Korean J Anesthesiol. **2022** Jun;75(3):255-265. doi: 10.4097/kja.21390. Epub 2021 Dec 29. PMID: 34963269
- Hussain N, Van den Langenbergh T, Sermer C, Fontes ML, Atrey A, Shaparin N, Sawyer TR, Vydyanathan A. Equivalent analgesic effectiveness between perineural and intravenous dexamethasone as adjuvants for peripheral nerve blockade: a systematic review and meta-analysis. **Can J Anaesth**. 2018 Feb;65(2):194-206. English. [doi: 10.1007/s12630-017-1008-8](https://doi.org/10.1007/s12630-017-1008-8). Epub 2017 Nov 21. PMID: 29164528

<https://static-content.springer.com/esm/art%3A10.1007%2Fs12630-017-1008-8/MediaObjects/12630_2017_1008_MOESM1_ESM.pdf>

- **Baeriswyl M, Kirkham KR, Jacot-Guillarmod A, Albrecht E.** Efficacy of perineural vs systemic dexamethasone to prolong analgesia after peripheral nerve block: a systematic review and meta-analysis. Br J Anaesth. 2017 Aug 1;119(2):183-191. [doi: 10.1093/bja/aex191](https://doi.org/10.1093/bja/aex191). PMID: 28854551.
- Chong MA, Berbenetz NM, Lin C, Singh S. Perineural Versus Intravenous Dexamethasone as an Adjuvant for Peripheral Nerve Blocks: A Systematic Review and Meta-Analysis. Reg Anesth Pain Med. 2017 May/Jun;42(3):319-326. [doi: 10.1097/AAP.0000000000000571](https://doi.org/10.1093/bja/aex191). PMID: 28252523.

Voir aussi la stratégie de

Pehora C, Pearson AM, Kaushal A, Crawford MW, Johnston B. Dexamethasone as an adjuvant to peripheral nerve block. Cochrane Database Syst Rev. 2017 Nov 9;11(11):CD011770. <https://doi.org/10.1002/14651858.cd011770.pub2>. PMID: [29121400](https://pubmed.ncbi.nlm.nih.gov/29121400/)

# Sources de données exploitées

<https://www.bium.ch/documents/bases-de-donnees/>

**Bases de données bibliographiques**

Medline ALL Ovid

Embase.com

Cochrane Central Register of Controlled Trials Wiley

**~~Registres d'enregistrements d'essais cliniques~~**

~~ClinicalTrials.gov~~

~~ICTRP - WHO International Clinical Trials Registry Platform~~

La recherche dans les registres d'enregistrements d'essais cliniques a été faite via Cochrane Central Registry of Controlled Trials

**Moteurs de recherche**

Google Scholar

# Vocabulaire

| **Concepts retenus** | Peripheral nerve block* | dexamethasone | Systemic (IV, IM) administration |
| --- | --- | --- | --- |
| **Termes libres** | ((nerve* OR conduction OR peripheral OR brachial OR cervical OR paracervical OR "quadratus lumborum" OR QL OR retrobulbar OR retroocular OR "transvers* abdom* plane" OR TAP OR fascia* OR pudendal OR "rectus sheath" OR Ilioinguinal OR iliohypogastric OR interscalene OR infraclavicular OR "infra clavicular" OR supraclavicular OR "supra clavicular" OR "supracapsular" OR "supra capsular" OR axillar* OR Intercostobrachial OR wrist OR "lumbar plexus" OR sciatic OR femoral OR adductor OR ankle OR saphenous OR popliteal OR obturator OR PENG OR interfascial OR paravertebral OR intercostal OR PECS OR "erector spinae" OR serratus) NEAR/6 block*)  ISB OR SSNB  "autonomic block*" OR "neurogenic block*"  "brachial plexus an$esthesia"  "cervical plexus an$esthesia"  "lumbar plexus an$esthesia"  "paracervical an$esthesia" OR "paracervical analgesia"  "retrobulbar an$esthesia"  ((shoulder OR ankle OR foot OR knee OR hip OR wrist OR hand OR "lower limb*" OR "upper limb*") NEAR/3 surg*):ab,ti,kw AND ('perineural drug administration'/exp OR (perineural* OR "Peri neural*"):ab,ti,kw) | Dexamethason* | intravenous*  IV  "I.V."  Venous  Intramuscular*  "intra muscular*"  IM  "I.M."  Systemic |
| **Emtree** | 'nerve block'/de OR 'brachial plexus anesthesia'/de OR 'cervical plexus block'/de OR 'intercostal nerve block'/de OR 'lumbar plexus block'/de OR 'paracervical block'/de OR 'quadratus lumborum block'/de OR 'retrobulbar anesthesia'/de OR 'transversus abdominis plane block'/de  ('joint surgery'/exp OR 'foot surgery'/exp OR 'hand surgery'/exp) AND ('perineural drug administration'/exp OR (perineural* OR "Peri neural*")ab,ti,kw) | 'dexamethasone'/de | 'dexamethasone'/exp/dd_iv 'intravenous drug administration'/de  'dexamethasone'/exp/dd_im OR 'intramuscular drug administration'/de |
| **MeSH** | "Nerve Block"/ OR "Autonomic Nerve Block"  (Exp Joints/su OR exp Lower Extremity/su OR exp Upper Extremity/su) AND (perineural* OR "Peri neural*").ab,ti,kf) | "Dexamethasone"/ | Exp "Administration, Intravenous"/  "Injections, Intramuscular"/ |

**Commentaires et Questions**

**Commentaires**

**Selon discussion du 22.05.2023**

- MeSH, "**Autonomic Nerve Block**" non gardés
- Termes Emtree: **'ganglion block'/exp** et **'stellate ganglion block'/de** non gardés

**Selon discussion du 04.07.2023**

Non conservés :

- 'regional anesthesia'/de ? et synonymes : "conduction an$esthesia" OR "region* an$esthesia'

"Anesthesia, Conduction"/

- decadron OR dexona
- parenteral
- 'intravenous regional anesthesia'/de - 'parenteral drug administration'/de

-'dexamethasone derivative'/exp pas de différence en concept quand ajouté aux concepts 2 et 3

- Filtres pop adultes : NON

- Ajouter perineural ? (synonymes ??) : NON

**05.07.2023**

- Ajout de termes en jaune selon l'article dans Uptodate pour décrire **peripheral** nerve block

<https://www.uptodate.com/contents/overview-of-peripheral-nerve-blocks?search=brachial%20plexus%20block&source=search_result&selectedTitle=4~43&usage_type=default&display_rank=4#H1772901492>

-A voir 1 conf abstracts non récupéré :

Golubovska I., Miscuks A., Kucina J. Analgesic effects of dexamethasone when given perineurally or intravenously in the upper arm bone fracture and shoulder joint surgery. Regional Anesthesia and Pain Medicine 2019 44:10 Supplement 1 (A212-) <http://dx.doi.org/10.1136/rapm-2019-ESRAABS2019.368>

Récupéré si ajout de

(('joint surgery'/exp OR 'foot surgery'/exp OR 'hand surgery'/exp OR ((shoulder OR ankle OR foot OR knee OR hip OR wrist OR hand OR "lower limb*" OR "upper limb*") NEAR/3 surg*):ab,ti,kw) AND perineural*)

**Décision 18.07.2023 : segment ajouté**

- Termes retenus pour l'équation Google Scholar **selon mail du 07.07.2023**

dexamethasone intravenous|intramuscular perineural nerve|peripheral|brachial|"quadratus lumborum"|interscalene|infraclavicular|supraclavicular|axillary|"lumbar plexus"|sciatic|femoral|adductor|ankle|saphenous|paravertebral|popliteal|obturator/PENG block

Termes retirés : cervical, paracervical, fasci et transversus.

Dans Cochrane, faut-il rajouter perineural pour les enregistrements d'essais cliniques : Oui **selon mail du 07.07.2023**

# Stratégies de recherche

## Medline ALL Ovid

1946 to July 17, 2023

**155 results on 18.07.2023**

*Filter adapted from Cochrane Highly Sensitive Search Strategy for identifying randomized trials in MEDLINE: sensitivity-maximizing version (2008 revision); Ovid format*

*See the Technical Supplement, Box 3c page 61:* [*https://training.cochrane.org/technical-supplement-chapter-4-searching-and-selecting-studies-v63*](https://training.cochrane.org/technical-supplement-chapter-4-searching-and-selecting-studies-v63)

("Nerve Block"/ OR "Autonomic Nerve Block"/ OR (((nerve* OR conduction OR peripheral OR brachial OR cervical OR paracervical OR "quadratus lumborum" OR QL OR retrobulbar OR retroocular OR "transvers* abdom* plane" OR TAP OR fascia* OR pudendal OR "rectus sheath" OR Ilioinguinal OR iliohypogastric OR interscalene OR infraclavicular OR "infra clavicular" OR supraclavicular OR "supra clavicular" OR "supracapsular" OR "supra capsular" OR axillar* OR Intercostobrachial OR wrist OR "lumbar plexus" OR sciatic OR femoral OR adductor OR ankle OR saphenous OR popliteal OR obturator OR PENG OR interfascial OR paravertebral OR intercostal OR PECS OR "erector spinae" OR serratus) ADJ6 block*) OR ISB OR SSNB OR "autonomic block*" OR "neurogenic block*" OR "brachial plexus an?esthesia" OR "cervical plexus an?esthesia" OR "lumbar plexus an?esthesia" OR "paracervical an?esthesia" OR "paracervical analgesia" OR "retrobulbar an?esthesia").ab,ti,kf. OR ((Exp Joints/su OR exp Lower Extremity/su OR exp Upper Extremity/su OR ((shoulder OR ankle OR foot OR knee OR hip OR wrist OR hand OR "lower limb*" OR "upper limb*") ADJ3 surg*).ab,ti,kf.) AND (perineural* OR "Peri neural*").ab,ti,kf.)) AND ('dexamethasone'/de OR (dexamethason*).ab,ti,kf.) AND (Exp "Administration, Intravenous"/ OR "Injections, Intramuscular"/ OR (intravenous* OR IV OR "I.V." OR venous OR intramuscular* OR "intra muscular*" OR IM OR "I.M." OR systemic).ab,ti,kf.) AND ((exp randomized controlled trial/ OR controlled clinical trial.pt. OR randomized.ab. OR randomised.ab. OR placebo.ab. OR drug therapy.fs. OR randomly.ab. OR trial.ab. OR groups.ab.) NOT (exp animals/ not humans.sh.))

## Embase.com

**417 results on 18.07.2023**

*Filter used : Glanville J, Foxlee R, Wisniewski S, Noel-Storr A, Edwards M, Dooley G. Translating the Cochrane EMBASE RCT filter from the Ovid interface to Embase.com: a case study. Health Info Libr J. 2019 Sep;36(3):264-277. doi: 10.1111/hir.12269. Updated version 30 April 2023*

[*https://sites.google.com/a/york.ac.uk/issg-search-filters-resource/home/rcts/embase-rct-filter*](https://sites.google.com/a/york.ac.uk/issg-search-filters-resource/home/rcts/embase-rct-filter)

*Recommanded by the Cochrane Handbook : see the Technical Supplement, Box 3e page 63 :* [*https://training.cochrane.org/technical-supplement-chapter-4-searching-and-selecting-studies-v63*](https://training.cochrane.org/technical-supplement-chapter-4-searching-and-selecting-studies-v63)

('nerve block'/de OR 'brachial plexus anesthesia'/de OR 'cervical plexus block'/de OR 'intercostal nerve block'/de OR 'lumbar plexus block'/de OR 'paracervical block'/de OR 'quadratus lumborum block'/de OR 'retrobulbar anesthesia'/de OR 'transversus abdominis plane block'/de OR (((nerve* OR conduction OR peripheral OR brachial OR cervical OR paracervical OR "quadratus lumborum" OR QL OR retrobulbar OR retroocular OR "transvers* abdom* plane" OR TAP OR fascia* OR pudendal OR "rectus sheath" OR Ilioinguinal OR iliohypogastric OR interscalene OR infraclavicular OR "infra clavicular" OR supraclavicular OR "supra clavicular" OR "supracapsular" OR "supra capsular" OR axillar* OR Intercostobrachial OR wrist OR "lumbar plexus" OR sciatic OR femoral OR adductor OR ankle OR saphenous OR popliteal OR obturator OR PENG OR interfascial OR paravertebral OR intercostal OR PECS OR "erector spinae" OR serratus) NEAR/6 block*) OR ISB OR SSNB OR "autonomic block*" OR "neurogenic block*" OR "brachial plexus an$esthesia" OR "cervical plexus an$esthesia" OR "lumbar plexus an$esthesia" OR "paracervical an$esthesia" OR "paracervical analgesia" OR "retrobulbar an$esthesia"):ab,ti,kw OR (('joint surgery'/exp OR 'foot surgery'/exp OR 'hand surgery'/exp OR ((shoulder OR ankle OR foot OR knee OR hip OR wrist OR hand OR "lower limb*" OR "upper limb*") NEAR/3 surg*):ab,ti,kw) AND ('perineural drug administration'/exp OR (perineural* OR "Peri neural*"):ab,ti,kw))) AND ('dexamethasone'/de OR (dexamethason*):ab,ti,kw) AND ('dexamethasone'/exp/dd_iv OR 'intravenous drug administration'/de OR 'dexamethasone'/exp/dd_im OR 'intramuscular drug administration'/de OR (intravenous* OR IV OR "I.V." OR venous OR intramuscular* OR "intra muscular*" OR IM OR "I.M." OR systemic):ab,ti,kw) AND (('randomized controlled trial'/exp OR (('controlled clinical trial'/de OR random*:ti,ab,tt OR 'randomization'/de OR 'intermethod comparison'/de OR placebo:ti,ab,tt OR (compare OR compared OR comparison):ti,tt OR ((evaluated OR evaluate OR evaluating OR assessed OR assess) AND (compare OR compared OR comparing OR comparison)):ab OR (open NEXT/1 label):ti,ab,tt OR ((double OR single OR doubly OR singly) NEXT/1 (blind OR blinded OR blindly)):ti,ab,tt OR 'double blind procedure'/de OR (parallel NEXT/1 group*):ti,ab,tt OR (crossover OR 'cross over'):ti,ab,tt OR ((assign* OR match OR matched OR allocation) NEAR/6 (alternate OR group OR groups OR intervention OR interventions OR patient OR patients OR subject OR subjects OR participant OR participants)):ti,ab,tt OR (assigned OR allocated):ti,ab,tt OR (controlled NEAR/8 (study OR design OR trial)):ti,ab,tt OR (volunteer OR volunteers):ti,ab,tt OR 'human experiment'/de OR trial:ti,tt) NOT ((((random* NEXT/1 sampl* NEAR/8 ('cross section*' OR questionnaire* OR survey OR surveys OR database or databases)):ti,ab,tt) NOT ('comparative study'/de OR 'controlled study'/de OR 'randomised controlled':ti,ab,tt OR 'randomized controlled':ti,ab,tt OR 'randomly assigned':ti,ab,tt)) OR ('cross-sectional study'/de NOT ('randomized controlled trial'/exp OR 'controlled clinical trial'/de OR 'controlled study'/de OR 'randomised controlled':ti,ab,tt OR 'randomized controlled':ti,ab,tt OR 'control group':ti,ab,tt OR 'control groups':ti,ab,tt)) OR ('case control*':ti,ab,tt AND random*:ti,ab,tt NOT ('randomised controlled':ti,ab,tt OR 'randomized controlled':ti,ab,tt)) OR (nonrandom*:ti,ab,tt NOT random*:ti,ab,tt) OR 'random field*':ti,ab,tt OR ('random cluster' NEAR/4 sampl*):ti,ab,tt))) NOT (('animal'/exp OR 'animal experiment'/exp OR 'animal model'/exp OR 'nonhuman'/exp) NOT 'human'/exp))

## Cochrane Central Register of Controlled Trials Wiley

Issue 7 of 12, July 2023

**304 results on 18.07.2023**

**#1:**

((((nerve* OR conduction OR peripheral OR brachial OR cervical OR paracervical OR "quadratus lumborum" OR QL OR retrobulbar OR retroocular OR (transvers* NEXT abdom* NEXT plane) OR TAP OR interscalene OR infraclavicular OR axillar* OR sciatic OR femoral OR adductor OR ankle OR saphenous OR fascia* OR pudendal OR "rectus sheath" OR Ilioinguinal OR iliohypogastric OR "infra clavicular" OR supraclavicular OR "supra clavicular" OR "supracapsular" OR "supra capsular" OR Intercostobrachial OR Wrist OR popliteal OR obturator OR PENG OR interfascial OR paravertebral OR intercostal OR PECS OR "erector spinae" OR serratus) NEAR/6 block*) OR ((autonomic OR neurogenic) NEXT block*) OR ((brachial OR cervical OR lumbar OR paracervical) NEXT plexus NEXT an*esthesia) OR "paracervical analgesia" OR (retrobulbar NEXT an*esthesia)):ab,ti,kw) AND ((dexamethason*):ab,ti,kw) AND ((intravenous* OR IV OR venous OR intramuscular* OR (intra NEXT muscular) OR IM OR systemic):ab,ti,kw) NOT ((Trial registry record):pt)

**#2 :**

((((nerve* OR conduction OR peripheral OR brachial OR cervical OR paracervical OR "quadratus lumborum" OR QL OR retrobulbar OR retroocular OR (transvers* NEXT abdom* NEXT plane) OR TAP OR interscalene OR infraclavicular OR axillar* OR sciatic OR femoral OR adductor OR ankle OR saphenous OR fascia* OR pudendal OR "rectus sheath" OR Ilioinguinal OR iliohypogastric OR "infra clavicular" OR supraclavicular OR "supra clavicular" OR "supracapsular" OR "supra capsular" OR Intercostobrachial OR Wrist OR popliteal OR obturator OR PENG OR interfascial OR paravertebral OR intercostal OR PECS OR "erector spinae" OR serratus) NEAR/6 block*) OR ((autonomic OR neurogenic) NEXT block*) OR ((brachial OR cervical OR lumbar OR paracervical) NEXT plexus NEXT an*esthesia) OR "paracervical analgesia" OR (retrobulbar NEXT an*esthesia) OR ((shoulder OR ankle OR foot OR knee OR hip OR wrist OR hand OR (lower NEXT limb*) OR (upper NEXT limb*)) NEAR/3 surg*)):ab,ti,kw) AND ((dexamethason*):ab,ti,kw) AND ((intravenous* OR IV OR venous OR intramuscular* OR (intra NEXT muscular) OR IM OR systemic):ab,ti,kw) AND ((perineural* OR (peri NEXT neural*)):ab,ti,kw)

**#1 OR #2**

## Google Scholar

**Export of the first 300 references using Harzing's Publish or Perish (Windows GUI Edition) on 18.07.2023**

dexamethasone intravenous|intramuscular perineural nerve|peripheral|brachial|"quadratus lumborum"|interscalene|infraclavicular|supraclavicular|axillary|"lumbar plexus"|sciatic|femoral|adductor|ankle|saphenous|paravertebral|popliteal|obturator|PENG block

# Résultats

**Date finale des recherches : 18.07.2023**

| **Database** | **Number of references** | |
| --- | --- | --- |
|  | **Found** | **After deduplication** |
| **Medline ALL Ovid**  1946 to July 17, 2023 | **155** | **155** |
| **Embase.com** | **417** | **286** |
| **Cochrane Central Register of Controlled Trials Wiley**  Issue 7 of 12, July 2023 | **304** | **97** |
| **Google Scholar** | **300** | **210** |
| **Total** | **1176** | **748** |

Les résultats ont été importés dans EndNote 20 et dédoublonnés avec Deduklick (Risklick AG).

Borissov N, Haas Q, Minder B, Kopp-Heim D, von Gernler M, Janka H, Teodoro D, Amini P. Reducing systematic review burden using Deduklick: a novel, automated, reliable, and explainable deduplication algorithm to foster medical research. Syst Rev. 2022 Aug 17;11(1):172. doi: 10.1186/s13643-022-02045-9. PMID: 35978441; PMCID: PMC9382798. <https://pubmed.ncbi.nlm.nih.gov/35978441/>

**Nombre de doublons identifiés automatiquement par Deduklick** **:** 427

**Nombre de doublons identifiés manuellement après Deduklick :** 1
